# Clinical and Autofluorescence Findings in Eyes with Pinguecula and Pterygium

**DOI:** 10.18502/jovr.v18i3.13773

**Published:** 2023-07-28

**Authors:** Amir-Hooshang Beheshtnejad, Hamed Ghassemi, Hossein Abdolkhalegh, Mehrnaz Atighehchian

**Affiliations:** ^1^Department of Ophthalmology, Farabi Eye Hospital, Tehran University of Medical Sciences, Tehran, Iran; ^2^Eye Research Center, Farabi Eye Hospital, Tehran University of Medical Sciences, Tehran, Iran

**Keywords:** Autofluorescence, Pinguecula, Pterygium

## Abstract

**Purpose:**

To assess the autofluorescence size and properties of pterygium and pinguecula by anterior segment autofluorescence (AS-AF) imaging and demonstrate the difference of autofluorescence size presented in AS-AF imaging compared to the extend size of the conjunctival lesion measured by anterior segment slit-lamp photography (AS-SLE).

**Methods:**

Twenty-five patients with primary pterygium and twenty-five with pinguecula were included in the study. In addition, 25 normal subjects were also enrolled as the control group. The AS-AF characteristics of pterygium and pinguecula lesions were analyzed. The size of lesions displayed in the AS-SLE photography versus the AS-AF images were also compared. AS-AF images were obtained using a Heidelberg retina angiograph which focused on the anterior segment. AS-SLE photography was acquired using a digital imaging system (BX900 HAAG-STREIT).

**Results:**

There were 44 (58.7%) male and 31 (41.3%) female patients; 19 (76%) and 20 (80%) patients had bilateral pterygium and pinguecula, respectively. All pinguecula lesions reflected hyperautofluorescence pattern in the AS-AF imaging. In 24 (96%) patients, the hyperautofluoresecence pattern was larger than the size of the clinical lesions displayed with the AS-SLE photography. Twenty-one (84%) patients with pterygium reflected a hyperautofluorescence pattern in AS-AF images; in one (4%) patient, the hyperautofluorescence pattern was larger than the clinical lesion size and four (16%) patients had no autofluorescence patterns in the AS-AF images. In the control group, in 14 (56%) subjects, a hypoautofluorescent pattern was revealed in the conjunctiva in AS-AF images. However, in 11 (44%) patients, hyperautofluorescence patterns were detected.

**Conclusion:**

AS-AF is a useful modality to monitor vascularization in conjunctival lesions. Pingueculae and pterygium show hyperautofluorescence in AS-AF imaging. The real size of the pinguecula lesions may be estimated with AS-AF characteristics, mostly presenting larger than the area size in AS-SLE photography. The autofluorescence size of the pterygium is smaller than the extent of visible pterygium in slit-lamp photography.

##  INTRODUCTION

A pterygium is an inflammatory ocular surface disease and triangular fibrovascular growth that emanates from the bulbar conjunctiva and crosses the cornea at the interpalpebral fissure on the nasal or temporal sides of the eye; mostly affecting the nasal rather than the temporal side.^[1,2,3,4]^

A pinguecula is a benign yellowish protruding lesion adjacent to the limbus that does not cross the cornea and may be visible on both sides of the cornea but is more often seen on the nasal side.^[5]^ The pathogenesis of these conjunctival lesions remains indistinctive. These degenerative conditions are affected by several intrinsic and extrinsic factors such as age, wind, solar, and ultraviolet radiation exposure.^[5,6]^ Fluorescence is the ability of specific molecules to radiate light energy of a longer wavelength when stimulated by a shorter wavelength light without injecting dye.^[7]^ Recently, fundus autofluorescence (AF) has been a beneficial imaging modality for the diagnosis of different retinal disorders. Retinal autofluorescence imaging is based on the stimulated emission of light from lipofuscin and is a noninvasive retinal pigment epithelium examination technique.^[7,8,9,10]^ The size of pinguecula can be evaluated by AF features, and often has a larger autofluorescence area size in the AF imaging than the visible lesion size in clinical examination by slit-lamp biomicroscopy.^[5,7]^ To the best of our knowledge, there have been a few reports on anterior segment autofluorescence (AS-AF) imaging for evaluating anterior segment disorders. The previous studies did not compare autofluorescence patterns between pterygium and pinguecula;^[7]^ therefore, in this study, we aimed to assess the discrepancy of AS-AF properties on these two wide-spread conjunctival diseases, and compare the AS-AF results to actual lesions' size displayed by slit-lamp photography.

**Figure 1:**
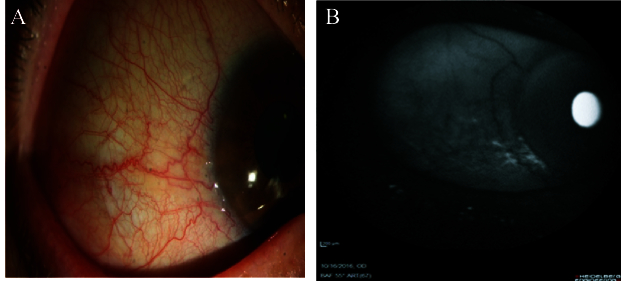
(A) Normal conjunctiva in slit-lamp photography. (B) Hypoautofluorescence pattern at the temporal area in AS-AF imaging.

**Figure 2:**
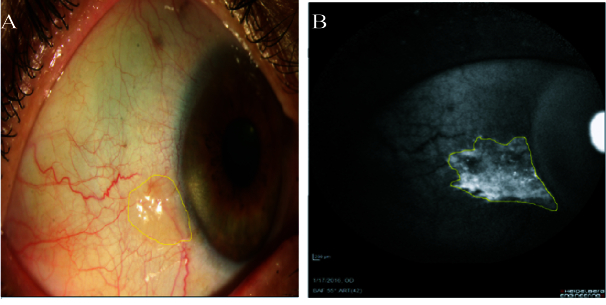
(A) Pinguecula at the temporal side in slit-lamp photography. (B) Hyperautofluoresecence pattern at the temporal side in AS-AF. The size of the autofluorescence area is larger than the size of the visible pinguecula in slit-lamp photography.

**Figure 3:**
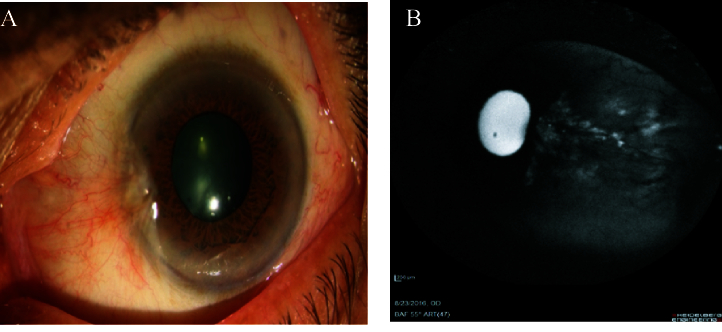
Pterygium at the nasal side of the left eye in slit-lamp photography. (B) Faint punctuate hyperautofluoresecence pattern is seen at the nasal side in AS-AF imaging. The size of the autofluorescence area of pterygium is smaller than the visible pterygium size in slit-lamp photography.

**Figure 4:**
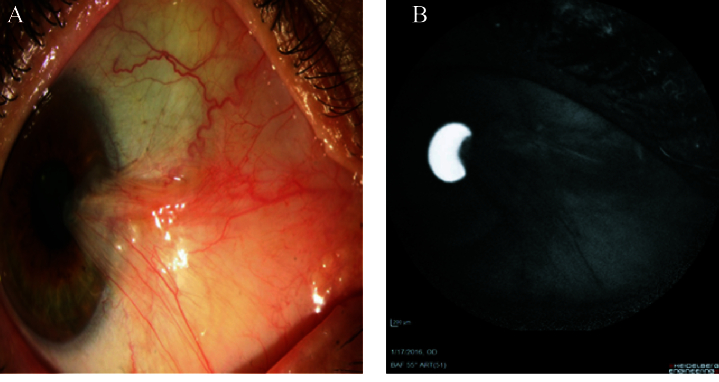
(A) Pterygium. (B) Complete hypoautofluorescence pattern in AS-AF imaging.

**Table 1:** The comparison of lesions size between slit-lamp photography and AS-AF imaging.


** Subjects **	**Mean slit-lamp photography area (mm^2^)**	**Mean AS-AF imaging ${\;}^{1}$ area (mm^2^)**	**Std. D^2^ slit-lamp photography area (mm^2^)**	**Std. D AS-AF imaging area (mm^2^)**	**Difference**	**95% Confidence Interval of the Difference**	***P*** **-value**
Pinguecula	10.128	19.320	4.149	7.795	–9.192	–11.446 to –6.937	< 0.001
Pterygium	50.924	7.194	21.286	5.461	43.730	33.570 to 53.889	< 0.001
Normal	0	0.388	0	0.526	–0.388	–0.605 to –0.170	0.001
	
	
AS-AF imaging, anterior segment autofluorescence imaging; mm^2^, square millimeter; Std*, *standard deviation *P*-value < 0.05							

##  METHODS

### Patients

The patients included in this study were those who visited the ocular surface eye clinic, Farabi Eye Hospital, Tehran University of Medical Sciences. Patients with pinguecula or primary pterygium with no other ocular surface disease were included in this study. Seventy-five patients (31 women, 44 men) between the ages of 21 and 73 years were enrolled, of whom 25 patients were diagnosed with pterygium and 25 were diagnosed with pingueculae. In addition, 25 healthy subjects with neither pterygium nor pinguecula were considered as a control group. Each patient underwent general ophthalmic examination including visual acuity assessment, slit-lamp biomicroscopy, intraocular pressure measurement with Goldmann applanation tonometry, and posterior segments examination with indirect ophthalmoscopy.

Inclusion criteria were confirmation of either having primary pterygium or pinguecula through slit-lamp biomicroscopy, and patients being older than 21 years. In bilateral cases, only one eye with primary pterygium with less inflammatory features and more advance stage was considered. Pterygium were classified into three stages according to the following method suggested by Yang et al – Stage I, the head of the pterygium does not reach the midline between the limbus and pupillary margin; Stage II, the head of the pterygium passes the midline but does not reach the pupil; and Stage III, the head of the pterygium passes the pupillary margin.^[1]^ The inflammation was clinically graded according to hyperemia in the site of pterygium excision zone as follows: 0 = none, 1 = mild, 2 = moderate, and 3 = severe.^[4]^

Exclusion criteria were a history of topical medication usage other than artificial tears one month before the study, recurrent pterygium, keratitis, atopic keratoconjunctivitis, inflammatory conjunctival disorders, previous ocular surface surgery, severe dry eye disease, abnormal eyelid function, conjunctival scarring, and symblepharon.

This study adhered to the tenets of the Declaration of Helsinki and the protocol of the study was approved by the Ethics Committee of Tehran University of Medical Sciences, Tehran, Iran; the approval number was IR.TUMS.VCR.REC.1396.4797. Informed consent was obtained from all patients and demographic characteristics of the patients including age, sex, family history, and, outdoor period time were considered.

### Slit-lamp Photography and AS-AF Image Techniques and Analysis

Anterior segment slit-lamp photography was obtained using a digital imaging system (BX900 HAAG-STREIT). A confocal scanning laser ophthalmoscope (Heidelberg Retina Angiography HRA2, Heidelberg) was used for the evaluation of anterior segment AF images. AS-AF imaging can be used for determining the size of conjunctival lesions such as pterygium and pinguecula. This device uses argon blue laser stimulating light with a 488 nm wavelength and a barrier filter that allows the passage of stimulating lights > 500 nm wavelength. The focus on the conjunctiva is obtained with the infrared mode of this device and AF images are saved in fluorescein angiogram mode without using fluorescein dye.^[7]^

In the present study, the pinguecula and pterygium lesions' size at the temporal or nasal of the cornea in slit-lamp photography was compared with the autofluorescence size of the lesions displayed in AS-AF. The autofluorescence region size in AS-AF was analyzed and measured in mm^2^ with IMAGE J software.

### Statistical Analysis

This study is a cross-sectional study. Statistical analysis was performed using SPSS software version 17. The correlation between the lesion size in AS-AF and slit-lamp photography was evaluated using a Pearson correlation test. Chi-square test was used for the study of the qualitative results and independent *t*-test was used for evaluating the quantitative findings. A *P*-value < 0.05 was considered as statistically significant. Discriminated analysis was used to evaluate the role of confounding factors.

##  RESULTS

A total of 75 patients were included in the study. Of these, 25 patients had primary pterygium and 25 had pinguecula. The remaining 25 patients neither had pterygium nor pinguecula and were considered as the control group.

On slit-lamp biomicroscopy, 24 (96%) patients had nasal pterygium and only 1 (4%) patient had temporal pterygium. Pinguecula was detected on the nasal and temporal sides in 13 (52%) and 12 (48%) patients, respectively. Moreover, 19 (76%) patients with pterygium and 20 (80%) patients with pinguecula had bilateral lesions. The mean age of patients was 46.89 ± 1.37 years (range: 21–73 years). Overall, 44 (58.7%) patients were males and 31 (41.3%) were females. There were 16 men and 9 women who had both pinguecula and pterygium.

While the prevalence of both lesions was slightly more in men than in women, there was no statically significant correlation between sex and presentation of these lesions (*P* = 0.41).

*Comparison of lesions' size between slit-lamp photography and AS-AF imaging*

A statistically significant correlation was observed between the lesions' size displayed in slit-lamp photography and the autofluorescence area size displayed in AS-AF imaging for pterygium and pinguecula.

In 25 patients with pinguecula, the mean size of pinguecula lesions in slit-lamp photography was 10.128 mm^2^ and the mean size of the autofluorescence area displayed through the AS-AF imaging was 19.32 mm^2^. The difference was –9.19 mm^2^ (95% CI: –11.44 to –6.94, *P* < 0.001). So, the mean size of the autofluorescence area displayed in AS- AF imaging was 9.19 mm^2^ larger than the mean size of the pinguecula displayed in slit-lamp photography (*P* = 0.001).

In 25 patients with pterygium, the mean size of the pterygium lesions displayed in slit-lamp photography versus the autofluorescence area displayed in AS-AF imaging was 50.92 mm^2^ and 7.19 mm^2^, respectively. The difference was 43.73 mm^2^ (95% CI: 33.57 to 53.88, *P* < 0.001). So, the mean size of the autofluorescence area displayed in the AS-AF imaging was 43.73 mm^2^ smaller than the mean size of the pterygium displayed in slit-lamp photography (*P* = 0.001).

In addition, in 14 (56%) participants with healthy conjunctiva and intact vessels who did not have either pinguecula or pterygium, their conjunctiva revealed a hypoautofluorescence pattern in the AS-AF images [Figure 1]. However, in 11 (44%) subjects, a hyperautofluorescence pattern was detected. The mean size of the autofluorescence area of the healthy conjunctiva was 0.388 mm^2^. The difference was 0.388 mm^2^ (95% CI: –0.60 to –0.17, *P* < 0.001). So, the mean size of the healthy conjunctiva reflected in the autofluorescence area in normal subjects in the AS-AF imaging was 0.388 mm^2^ larger than the normal conjunctiva images reflected in slit-lamp photography (*P* = 0.001).

Table 1 shows the comparison of lesion sizes displayed between slit-lamp photography and AS-AF imaging.

*Comparative images between slit-lamp photography and AS-AF imaging*

The pinguecula lesions were revealed as hyperautofluorescence pattern in the AS-AF images in all patients. This well-defined autofluorescence area in 24 (96%) patients displayed a greater size than the visible part extension of pinguecula lesions displayed on slit-lamp photography [Figure 2].

The pterygium lesions were revealed as hyperautofluorescence patterns in the AS-AF images of 21 (84%) patients. This autofluorescence area displayed a greater size than the pterygium size displayed in slit-lamp photography in one (4%) patient [Figure 3]. On the other hand, four (16%) patients with pterygium did not demonstrate an autofluorescence pattern and were reflected completely as hypoautofluorescence pattern in the AS-AF images [Figure 4].

##  DISCUSSION

Pinguecula is a common conjunctival disease and pterygium is another benign conjunctival disorder that usually grows from the bulbar conjunctiva and invades the corneal surface. These degenerative disorders typically affect the interpalpebral conjunctiva, mostly the nasal side.^[1,2,3,4][11]^ Sun (ultraviolet light) exposure and exposure to environmental factors such as wind and dust increase the risk of these ocular surface diseases.^[12,13,14,15,16]^ Huseyin Dundar et al investigated the effects of using soft contact lenses on pinguecula prevalence.^[6]^

Recently, AF was introduced as a noninvasive imaging modality for the diagnosis and follow-up of several retinal disorders. ${\;}^{\left[9--10,17--19\right]}$ The AF is obtained by the 488 nm stimulating wavelength light argon blue laser and with a barrier filter at 500 nm wavelength, which suppresses the excitation light. This barrier filter allows the passage of wavelengths of > 500 nm. Short-wavelength excited signals are mainly derived from the RPE lipofuscin.^[7,9,10]^ It is possible to capture anterior segment images with the infrared process of this modality, where AS-AF images are then registered in fluorescein angiography mode, without using fluorescein dye.^[7,8,9]^

To the best of our knowledge, few studies have discussed the use of AS-AF imaging for determining pinguecula or pterygium size. Moreover, limited studies showed pinguecula had larger diffuse or punctuate autofluorescence patterns than the clinical lesions size.^[5,7]^ Our study is based on the evaluation of the autofluorescence size and pattern of pinguecula and pterygium by AS-AF imaging. It also shows the comparison of visible conjunctival lesion sizes displayed in slit-lamp photography.

As the present study showed, in most patients with pinguecula, the AF pattern was punctate hyperautofluorescence which showed greater size than the clinical features as revealed through slit-lamp photography. On the other hand, in most patients with pterygium, the AF pattern was hyperautofluorescence which had a smaller size than the visible lesion size reflected in slit-lamp photography. It is noted that the pinguecula lesions were characterized by hyperautofluorescence patterns with greater size than the clinical features of the actual lesion while pterygium lesions were displayed as hyperautofluorescence patterns with a smaller size than the clinical features of the actual lesions. Moreover, the AS-AF imaging of 16% of patients with pterygium did not display autofluorescence patterns, whereas the AS-AF imaging of 44% of patients with normal conjunctiva displayed a small-size hyperautofluorescence pattern.

Hence, this study shows that although early inflammatory damages of pinguecula and pterygium may not be detected in clinical examinations, AS-AF via autofluorescence changes can show early signs of inflammatory damage before clinical presentation.

AF imaging can reveal any changes in various retinal diseases based on RPE lipofuscin deposits.^[10]^ These lipofuscin granules contain specified fluorophores that are responsible for creating FAF imaging.^[5,7]^ The lipofuscin accumulation may be associated with conjunctival degenerative disorders and some evidence showed oxidative damage plays an important role in lipofuscin genesis.^[20]^ Therefore, the hyper-AF pattern in pinguecula may originate from lipofuscin granules. However, in pterygium lesions, the reduction or absence of autofluorescence reflectivity may depend on the decreased level of lipofuscin granules or some fibrovascular blockage. Hence, Further studies with histopathological and molecular complimentary evaluations are needed to confirm this hypothesis.

In summary, normal conjunctiva with healthy epithelial surface and intact vascular pattern reveals a hypoautofluorescence pattern in AS-AF imaging; however, a hyperautofluorescence pattern may suggest the early stage of a conjunctival disorder. Therefore, this imaging modality can be beneficial for detecting the pathological changes in the early stages of the conjunctival diseases including pinguecula and pterygium.

##  Financial Support and Sponsorship

None.

##  Conflicts of Interest

None.

## References

[B1] Kim YJ, Yoo SH, Chung JK (2014). Reconstruction of the limbal vasculature after limbal-conjunctival autograft transplantation in pterygium surgery: An angiography study. Invest Ophthalmol Vis Sci.

[B2] Gulkilik G, Kocabora S, Taskapili M, Ozsutcu M (2006). A new technique for pterygium excision: air-assisted dissection. Ophthalmologica.

[B3] Akbari M, Soltani-Moghadam R, Elmi R, Kazemnejad E (2017). Comparison of free conjunctival autograft versus amniotic membrane transplantation for pterygium surgery. J Curr Ophthalmol.

[B4] Ghoz N, Elalfy M, Said D, Dua H

[B5] Kim TH, Chun YS, Kim JC (2013). The pathologic characteristics of pingueculae on autofluorescence images. Korean J Ophthalmol.

[B6] Dundar H, Kocasarac C (2019). Relationship between contact lens and pinguecula. Eye Contact Lens.

[B7] Utine CA, Tatlipinar S, Altunsoy M, Oral D, Basar D, Alimgil LM (2009). Autofluorescence imaging of pingueculae. Br J Ophthalmol.

[B8] Spaide RF (2003). Fundus autofluorescence and age-related macular degeneration. Ophthalmology.

[B9] Boon CJ, Jeroen Klevering B, Keunen JE, Hoyng CB, Theelen T (2008). Fundus autofluorescence imaging of retinal dystrophies. Vision Res.

[B10] Sepah YJ, Akhtar A, Sadiq MA, Hafeez Y, Nasir H, Perez B, et al (2014). Fundus autofluorescence imaging: Fundamentals and clinical relevance. Saudi J Ophthalmol.

[B11] Zhao F, Cai S, Huang Z, Ding P, Du C (2020). Optical coherence tomography angiography in pinguecula and pterygium. Cornea.

[B12] Yazar S, Cuellar-Partida G, McKnight CM, Quach-Thanissorn P, Mountain JA, Coroneo MT, et al (2015). Genetic and environmental factors in conjunctival UV autofluorescence. JAMA Ophthalmol.

[B13] Wolffsohn JS, Drew T, Sulley A (2014). Conjunctival UV autofluorescence--Prevalence and risk factors. Cont Lens Anterior Eye.

[B14] Elhamaky TR, Elbarky AM (2018). Outcomes of vertical split conjunctival autograft using fibrin glue in treatment of primary double-headed pterygia. J Ophthalmol.

[B15] Jiang J, Gong J, Li W, Hong C

[B16] Hueber A, Grisanti S, Diestelhorst M (2005). Photodynamic therapy for wound-healing modulation in pterygium surgery. A clinical pilot study Graefes Arch Clin Exp Ophthalmol.

[B17] McBain VA, Townend J, Lois N (2007). Fundus autofluorescence in exudative age-related macular degeneration. Br J Ophthalmol.

[B18] Schmitz-Valckenberg S, Pfau M, Fleckenstein M, Staurenghi G, Sparrow JR, Bindewald-Wittich A, et al (2021). Fundus autofluorescence imaging. Prog Retin Eye Res.

[B19] Holz FG, Steinberg JS, Göbel A, Fleckenstein M, Schmitz-Valckenberg S (2015). Fundus autofluorescence imaging in dry AMD: 2014 Jules Gonin lecture of the Retina Research Foundation. Graefes Arch Clin Exp Ophthalmol.

[B20] Davies S, Elliott MH, Floor E, Truscott TG, Zareba M, Sarna T, et al

